# Genome-Scale Mutational Analysis of Cathode-Oxidizing *Thioclava electrotropha* ElOx9^T^

**DOI:** 10.3389/fmicb.2022.909824

**Published:** 2022-06-10

**Authors:** Joshua D. Sackett, Nitin Kamble, Edmund Leach, Taruna Schuelke, Elizabeth Wilbanks, Annette R. Rowe

**Affiliations:** ^1^Department of Biological Sciences, University of Cincinnati, Cincinnati, OH, United States; ^2^Department of Ecology, Evolution, and Marine Biology, University of California, Santa Barbara, Santa Barbara, CA, United States

**Keywords:** extracellular electron transfer (EET), lithotrophy, Tn-seq, marine bacterium, marine sediment, electrotroph, genomics

## Abstract

Extracellular electron transfer (EET) – the process by which microorganisms transfer electrons across their membrane(s) to/from solid-phase materials – has implications for a wide range of biogeochemically important processes in marine environments. Though EET is thought to play an important role in the oxidation of inorganic minerals by lithotrophic organisms, the mechanisms involved in the oxidation of solid particles are poorly understood. To explore the genetic basis of oxidative EET, we utilized genomic analyses and transposon insertion mutagenesis screens (Tn-seq) in the metabolically flexible, lithotrophic Alphaproteobacterium *Thioclava electrotropha* ElOx9^T^. The finished genome of this strain is 4.3 MB, and consists of 4,139 predicted ORFs, 54 contain heme binding motifs, and 33 of those 54 are predicted to localize to the cell envelope or have unknown localizations. To begin to understand the genetic basis of oxidative EET in ElOx9^T^, we constructed a transposon mutant library in semi-rich media which was comprised of >91,000 individual mutants encompassing >69,000 unique TA dinucleotide insertion sites. The library was subjected to heterotrophic growth on minimal media with acetate and autotrophic oxidative EET conditions on indium tin oxide coated glass electrodes poised at –278 mV vs. SHE or un-poised in an open circuit condition. We identified 528 genes classified as essential under these growth conditions. With respect to electrochemical conditions, 25 genes were essential under oxidative EET conditions, and 29 genes were essential in both the open circuit control and oxidative EET conditions. Though many of the genes identified under electrochemical conditions are predicted to be localized in the cytoplasm and lack heme binding motifs and/or homology to known EET proteins, we identified several hypothetical proteins and poorly characterized oxidoreductases that implicate a novel mechanism(s) for EET that warrants further study. Our results provide a starting point to explore the genetic basis of novel oxidative EET in this marine sediment microbe.

## Introduction

The discovery of extracellular electron transfer (EET) – the process by which microorganisms transfer electrons across their membrane(s) to/from solid-phase materials – has important implications for a wide range of biogeochemically important processes (Gralnick and Newman, 2007; Richter et al., 2012). Though our understanding of EET mechanisms is limited to just a few model mineral-reducing microbes [such as *Shewanella oneidensis* MR-1 (Lies et al., 2005; Nevin and Lovley, 2010), *Geobacter metallireducens* (Reguera et al., 2005; Reguera and Kashefi, 2019), *Pseudomonas aeruginosa* (Shen et al., 2014)], there is a growing diversity of microorganisms and physiologies that are thought to be capable of extracellular electron transfer, including phototrophs (Bose et al., 2014; Guzman et al., 2019; Gupta et al., 2020), sulfur and iron oxidizing microorganisms (Myers and Nealson, 1988; Bond and Lovley, 2003; Reguera et al., 2005; Rowe et al., 2015), anerobic ammonia oxidizers (Qu et al., 2014; Shaw et al., 2020) and consortia such as anaerobic methane oxidizers (Villano et al., 2010; Scheller et al., 2016; Gao et al., 2017) and syntrophic ethanol oxidizers (Rotaru et al., 2014). These observations span marine and freshwater systems and suggest that we are barely scratching the surface of the role EET plays in microbial metabolisms and the role of these processes in many environmental systems.

Though the role of EET in iron oxidation has recently been identified (Jiao and Newman, 2007; Liu et al., 2012; Summers et al., 2013; Bose et al., 2014; Barco et al., 2015), the mechanisms of *oxidation* of other inorganic and often insoluble materials, such as sulfur minerals, are poorly understood (Emerson, 2012). As the proteins involved in these reactions remain unknown, many omics-based studies fail to identify them in datasets, leaving them to accumulate among the conserved/hypothetical genes of unknown function, which are prevalent in environmental omics surveys. Coupled with the fact that these physiologies are difficult to detect in nature via chemistry alone (whether due to low substrate concentrations, the transient nature of reactants and/or products, or competition with abiotic or geochemical reactions), our understanding of the role these processes play in many environmental systems is severely limited (Ilbert and Bonnefoy, 2013). This is especially true for subseafloor marine sediments, which account for an estimated 2.9 × 10^29^ microbial cells and ∼0.6% of Earth’s total biomass (Kallmeyer et al., 2012). Currently, direct measurements of microbial metabolism in the “deep” and “dark” oceans far exceed the expected models based on the influx of organic carbon into the system (Burd et al., 2010). Metabolisms, such as lithotrophy and/or lithoautrophy, which depend less on organic carbon and may actually act as a source for organic carbon, may account for at least some of the missing carbon in current global carbon budgets (Swan et al., 2011). Therefore, the identification of genes to aid in understanding and detection of these processes in nature is crucial.

A growing number of studies over the past decade have expanded our knowledge of the diversity of marine microorganisms that engage in EET in marine sediments (Rowe et al., 2015; Chang et al., 2018; Lam et al., 2018, 2019). *Thioclava electrotropha* ElOx9^T^, originally isolated from marine sediment bioelectrochemical enrichments from Catalina Island, California, is one such bacterium (Rowe et al., 2015; Chang et al., 2018). Species of *Thioclava* are widespread in marine sediments and surface waters (Sorokin et al., 2005; Rowe et al., 2015; Liu et al., 2017; Chang et al., 2018; Karbelkar et al., 2019), and several strains are known lithoautotrophs capable of sulfur oxidation. Strain ElOx9^T^ is metabolically flexible, capable of oxygen or nitrate respiration, heterotrophic growth with acetate or glucose, and autotrophic growth with H_2_ or reduced sulfur substrates (S^2–^, S^0^, or S_2_O_3_^2–^) as energy sources (Chang et al., 2018). Recent bioelectrochemical investigations into the EET phenotype in *T. electrotropha* ElOx9^T^ indicate that biofilms that form on poised cathodes are electrochemically active (Rowe et al., 2015; Karbelkar et al., 2019) and that electron transfer is facilitated by direct contact – not mediated by soluble electron carriers – between the biofilm and the electrode surface under both aerobic (Rowe et al., 2015) and anaerobic conditions (Karbelkar et al., 2019). However, our preliminary genomic investigations failed to identify highly probable candidate genes that could be involved in EET, and consequently, the genetic basis of this process has yet to be determined.

Transposon saturation mutagenesis sequencing methods, collectively termed Tn-seq, have emerged as powerful, high-throughput tools to generate and test genome-scale mutant libraries and define gene essentiality under various growth conditions (Jacobs et al., 2003; Cameron et al., 2008; van Opijnen et al., 2009). These methods rely on the generation and pooling of a dense transposon library in the organism of interest in which the total number of mutants generated approximates the number of theoretically possible transposon insertion sites (Freed, 2017). This pooled mutant library (parent library) is then subjected to a growth condition of interest. Genomic DNA from the parent library and from all experimental growth conditions is isolated, transposon-genome junctions are sequenced, and the location and frequency of transposon insertions throughout the genome are determined. Essentiality and fitness determinations can be made based on the frequency of gene insertions in a particular library or by comparing insertion frequencies between differential growth conditions (i.e., comparing before and after a growth selection). This method has been employed to identify essential genes in diverse microorganisms, such as *Mycoplasma genitalium* (Glass et al., 2006), *Vibrio cholerae* (Cameron et al., 2008), *Rhodopseudomonas palustris* (Pechter et al., 2016), *G. sulfurreducens* (Rollefson et al., 2009; Chan et al., 2017), and *S. oneidensis* MR-1 (Bouhenni et al., 2005; Brutinel and Gralnick, 2012; Yang et al., 2014; Baym et al., 2016; Rowe et al., 2021). From Tn-seq experiments conducted in *G. sulfurreducens*, Chan et al. (2017) identified a putative methyl-accepting chemotaxis-cyclic dinucleotide sensing network (*esnABCD*) required for electrode colonization and a putative porin-cytochrome conduit complex (*extABCD*) required for growth with electrodes. In *S. oneidensis* MR-1, Rowe et al. (2021) identified and bioelectrochemically characterized five genes involved in EET: a putative diguanylate cyclase involved in both reductive and oxidative EET, and four other genes that play significant roles just in oxidative EET. These studies highlight the utility of Tn-seq experiments in identifying genes involved in physiologic processes on a genome scale and in an efficient, high-throughput manner.

To investigate the genetic basis of oxidative EET in ElOx9^T^, we coupled comprehensive genomic analysis with genome-scale transposon insertion mutagenesis sequencing screens to identify genes deemed essential for (1) heterotrophic growth in semi-rich media, (2) heterotrophic growth in minimal media with acetate (also used for electrode pre-growth), and (3) autotrophic oxidative EET conditions on an indium tin oxide (ITO) coated glass electrode poised at –278 mV vs. SHE or un-poised for open circuit conditions for comparative analysis.

## Materials and Methods

### Bacterial Strains and Culture Conditions

All cultures used in this work were stored in 40% glycerol at –80°C unless otherwise stated. Prior to each experiment, *T. electrotropha* ElOx9^T^ cultures were grown from a single colony isolated on either Difco™ Marine (DM) broth or Luria-Bertani (LB) broth with added NaCl and other ions (LBS + Ions) as described previously (Chang et al., 2018), with the addition of 1.5% agar. *E. coli* WM3064, a diaminopimelic acid (DAP) auxotroph, was utilized as a donor for conjugation and routinely grown on LB with the addition of 300 μM DAP. Kanamycin (100 mg/L) was routinely used to select for plasmid or transposon insertions in respective strains. All cultures were grown at 30°C in liquid media unless otherwise stated. Though ElOx9^T^ strains were grown in the enriched media DM and LBS + Ions for maintenance, a low-sulfate saltwater base (SWB) minimal medium (Rowe et al., 2015) was used for the majority of growth and electrochemical experiments. For heterotrophic growth, 5 mM sodium acetate was added as an electron donor.

### Genomic DNA Isolation, Sequencing, and Analysis

Genomic DNA was isolated from turbid cultures with the DNeasy PowerSoil Kit (Qiagen, Germantown, MD) according to the manufacturer’s instructions. DNA libraries were prepared for Illumina sequencing as described previously (Chang et al., 2018). DNA for long read sequencing was extracted using the Qiagen Blood and Tissue Kit (Qiagen, Germantown, MD). The DNA extract was barcoded using the Native Barcoding Kit 1D (Oxford Nanopore, United Kingdom) and prepared for sequencing using the Ligation Sequencing Kit 1D. NanoPore libraries were demultiplexed and base called with MinKNOW software (V.2). The genome was first assembled with Flye v. 2.7 (Kolmogorov et al., 2019) using the NanoPore long-read sequences. Illumina reads were aligned to the genome using Burrows–Wheeler aligner (Li and Durbin, 2010) and subsequently used to polish the long-read assembly using Pilon v. 1.23 (Walker et al., 2014) using default parameters.

Genome statistics were determined with QUAST v4.4 (Gurevich et al., 2013), and assembly quality was assessed with CheckM v.1.0.18 (Parks et al., 2015). The genome was annotated using the NCBI Prokaryotic Genome Annotation Pipeline (PGAP). Potential metabolic pathways were identified using KEGG’s BlastKOALA functional characterization tool (Kanehisa et al., 2016). Protein-encoding genes were analyzed for the presence of carbohydrate-active enzymes (CAZymes; Lombard et al., 2014) using the dbCAN2 automated carbohydrate-active enzyme annotation server (Yin et al., 2012; Zhang et al., 2018). Proteins annotated as CAZymes by a minimum of two independent methods (any combination of pHMMER [hidden Markov model-based annotation], DIAMOND (Buchfink et al., 2014) [sequence alignment-based annotation], or Hotpep (Busk et al., 2017)[peptide pattern recognition-based annotation]) were considered positive hits. Putative heme-binding proteins were identified by creating a searchable database of ElOx9^T^’s genome in Prosite (Hulo et al., 2006) and searching the database for c-type cytochrome heme-binding motifs (CX_2–4_CH). PSORTb v 3.0.3 (Yu et al., 2010) was subsequently used to predict the localization of those proteins. The nearest neighboring genomes were identified with the Genome Taxonomy Database (GTDB-tk v 1.7.0; Chaumeil et al., 2020). Whole genome average nucleotide identity (ANI) values between ElOx9^T^ and neighbors identified by GTDB-tk were calculated with the Kostas lab ANI calculator (Rodriguez-R and Konstantinidis, 2016).

### Transposon Mutant Library Generation and Selection

Transposon mutagenesis was performed by conjugal transfer of the modified *mariner* transposon pEB001 (Brutinel and Gralnick, 2012) to ElOx9^T^ from *Escherichia coli* WM3064 as described previously (Chan et al., 2017). In brief, this involved conjugation of the WM3064 donor strain containing pEB001 with ElOx9^T^ on the permissive media LBS + Ions agar containing 300 μM DAP. Pre-conjugation cultures of *E. coli* and ElOx9^T^ were grown at 30°C for 12–16 h and washed 3 × with LBS + Ions via centrifugation and resuspension. ElOx9^T^ cells were heat shocked in a pre-heated water bath (45°C for 5 min) and conjugation was performed by combining WM3064 and ElOx9^T^ to a final OD_600_ ratio of 1:0.65 in 100 μl of LBS + Ions and spotted in 10 μl aliquots onto permissive media. Negative controls were performed using LBS + Ions (DAP-) plates for WM3064 and LBS + Ions + Kan plates for ElOx9^T^. Conjugation plates were incubated for 12–16 h. These conjugation spots were collected using a nichrome wire loop washed 3X in 1X phosphate buffer and diluted onto large (200 mm diameter) LBS + Ions + Kan agar plates. Plates were incubated at 30°C for 16–24 h. until visible colonies formed. Successful conjugation was determined using colony PCR on select colonies, targeting the mechanosensitive conductance channel mscL in ElOx9^T^ (absent in WM3064, primers mscL_Fwd and mscL_Rev) and the inserted transposon (primers ARR_Tn_Fwd and ARR_Tn_Rev). Primer sequences are listed in Supplementary Table 1. Approximately 9.1 × 10^4^ colonies were generated, pooled in approximately 10 mL of LBS + Ions with 10% glycerol, and 1 mL aliquots were stored at –80°C for use in Tn-seq selection experiments.

ElOx9^T^ library transconjugants (containing a viable insertion of the *mariner* transposon) from these frozen stocks were sequenced directly (hereafter referred to as the LBS + Ions parent library). Triplicate libraries were grown in SWB + acetate for 16 h and growth was monitored by OD_600_ to ensure at least six doublings occurred. Cells from SWB + acetate were harvested for sequencing. Pre-growth for the electrochemical systems (hereafter referred to as “Pre-Electrochemistry”) was treated in the same way, however, aliquots of the cultures (10–20% of total culture volume) were added to bioelectrochemical systems at an OD_600_ of 0.2 for both poised potential and open circuit conditions as described below. The remaining cells from Pre-Electrochemistry growth were collected for sequencing. Electrochemical “growth” was calculated to be an average of 6 generations after 6 days. This was calculated from an autotrophic hydrogen growth curve of ElOx9^T^ that consumes 2.1 C based on cell yields per electron. For current ranging from 3 to 6 μA, the range of coulombs consumed at ∼6 days is between 1.5 and 3 C. This was in the desired range for the lowest current consuming experiments.

### Bioelectrochemical Measurements

The electrochemical analysis of the ElOx9^T^ library was performed in standard three-electrode bioreactors as described previously (Rowe et al., 2015). An ITO coated glass (Delta Technologies, Loveland, CO) with a 10.68 cm^2^ surface area was used as a working electrode, platinum wire (Sigma-Aldrich, St. Louis, MO) as a counter electrode, and Ag/AgCl (1M KCl; CH Instruments, Austin, TX, United States) as a reference electrode. The reactors were inoculated with 30 ml of SWB media and constantly purged with 0.2 μm-filtered ambient air to provide O_2_. Chronoamperometry and cyclic voltammetry (CV) analyses were performed using a 16-channel potentiostat (Biologic, Seyssinet-Pariset, France). For Chronoamperometry studies, the working electrode was poised at –278 mV vs. SHE to act as the electron donor for the cells. For CV, the working potential was cycled between –378 and 622 mV vs. SHE at a scan rate of 1 mV/s. The reactors were inoculated with a resuspended cell culture from the pre-growth to a final OD_600_ of 0.2 once a stable baseline current was achieved. Analysis of the electrode-attached biofilms was performed after the cathodic current in the bioreactor maximized and a CV was recorded. As kill controls were not permissible due to the need to outgrow electrode-attached cells, CVs were compared to separate experiments with wild-type ElOx9^T^ in which CVs were recorded before and after the addition of the ubiquinone mimic Antimycin A (50 μM final concentration) to discern biologically mediated EET processes from abiotic processes. The addition of Antimycin A results in a strong reduction in current consumption and reduced catalytic activity as seen in the CVs (Supplementary Figure 4). Open circuit conditions were run identically to poised potential conditions; however, no voltage was applied. Following electrochemical experiments, planktonic cells and spent cathode media were removed from all reactors, and libraries were outgrown on SWB + acetate at 30°C prior to collection of biomass for sequencing. All planktonic- and biofilm-associated cells (recovered via scraping) from the outgrowths were collected for sequencing.

### Transposon Library Sequencing

Genomic DNA from Tn-seq selection conditions and parent libraries was isolated with the Wizard Genomic DNA Purification Kit (Promega, Madison, WI). To isolate transposon insertion sites, up to 3 μg of genomic DNA was digested with *Mme*I (New England Biolabs, Ipswich, MA), heat inactivated and treated with Antarctic phosphatase (New England Biolabs, Ipswich, MA). The *Mme*I digested and Antarctic phosphatase-treated genomic DNA fragments were end repaired and dA-tailed with the NEBNext Ultra II End Repair/dA-Tailing Module (New England Biolabs, Ipswich, MA). Illumina adaptors (NEBNext Multiplex Oligos for Illumina Dual Index Primers Set 1, New England Biolabs, Ipswich, MA) were then ligated to these fragments with the NEBNext Ultra II Ligation Module (New England Biolabs, Ipswich, MA). These reactions were treated with USER enzyme (New England Biolabs, Ipswich, MA) and purified using the GeneJET Gel Extraction Kit (Thermo Fisher Scientific, Waltham, MA) and eluted in 50 μl prior to quantification using the Qubit dsDNA BR Assay Kit and a Qubit 4 Fluorometer (Thermo Fisher Scientific, Waltham, MA).

Approximately 50 ng of purified transposon-genome junction DNA was used in PCR enrichments using Phusion High-Fidelity DNA polymerase with GC buffer master mix (Thermo Fisher Scientific, Waltham, MA), custom forward primer ARR_Read1_TnF, and NEBNext Index reverse primers from the NEBNext Multiplex Oligos for Illumina Dual Index Primers Set 1 kit (New England Biolabs, Ipswich, MA) that anneal to the inverted repeat of the transposon and the ligated Illumina adaptor, respectively. Primer sequences are shown in Supplementary Table 1. PCR reactions were then run on 2% agarose gel. Expected PCR bands of ∼174 bp were excised from the gel cleaned using the GeneJET Gel Extraction kit (Thermo Fisher Scientific, Waltham, MA). PCR products were sent to Novogene (Sacramento, CA) for sequencing, following a quality control test using Bioanalyser traces. Individually barcoded libraries were pooled following the scheme in Supplementary Table 1 and sequenced in two lanes on an Illumina HiSeq 2500 with 2 × 150 paired-end chemistry. Manufacturers’ protocols were followed for all steps outlined above unless otherwise stated.

### Transposon Insertion Mutagenesis Screens Data Analysis

Analysis of Tn-seq data was performed using high performance computing resources provided by the Ohio Supercomputer Center (Ohio Supercomputer Center, 1987). Forward reads were truncated at the start of the NEBNext adaptor using Trimmomatic v. 0.36 (Bolger et al., 2014) allowing for up to two mismatches over the length of the adaptor sequence, reverse complemented using FASTX-Toolkit (Gordon and Hannon, 2010), reverse complements were truncated at the beginning of the *Mme*I recognition site endogenous to the transposon with Trimmomatic v. 0.36 (allowing for two mismatches over the length of the inverted repeat – *Mme*I sequence), and reverse complemented back to + sense. Upon removal of four bases belonging to the transposon’s inverted repeat (between *Mme*I recognition site and genomic DNA) and considering the end repair step removes the two bp 5’ overhang following *Mme*I digestion, the expected genomic DNA insert size was 14 bp. Reads were then filtered to retain genomic DNA inserts beginning with “TA” and ranging from 14 to 18 bp using awk. Remaining reads were then quality filtered using Trimmomatic with the following options: SLIDINGWINDOW:1:20 and MINLEN:14. Reverse reads were excluded from the analysis due to ambiguous base calls in the genomic DNA insert that, when removed, resulted in insert lengths < 14 bp.

Subsequent steps were conducted using a combination of publicly available programs and scripts from https://github.com/jbadomics/tnseq. Filtered reads were mapped to the ElOx9^T^ genome and plasmid with Bowtie1 v. 1.1.2 (Langmead et al., 2009), discarding reads without 100% identity to a unique position in the genome. Insertion sites were formatted using bioawk and insertions per gene were tabulated with the tabulate_insertions.py python script. Further, insertion events that occurred in the last 5% of the gene sequence were omitted as Yang et al. (2014) found higher transposon insertion frequencies in essential genes within the last ∼2% of the gene sequence, suggesting that insertions at the end of the gene may not be as effective in disrupting gene function. Genes lacking TA sites and genes for which less than half of the total number of TA sites within the gene are unique were excluded from our analysis. To account for differences in sequencing depth, reads per kilobase (RpK) values were normalized to 10^7^ reads per sample based on the total number of reads that mapped to a single TA site in the genome and plasmid, as described previously (Pechter et al., 2016).

### Determination of Gene Essentiality

Gene essentiality calls were determined following a similar statistical approach reported previously (Pechter et al., 2016). For each replicate, log_2_[normalized RpK] values were analyzed via histogram. Outliers were removed, the mean and standard deviation of the distribution were calculated, and a normal distribution curve was fitted to the data using the mean and SD. Essential genes were those with log_2_[normalized RpK] values less than 3 SD below the mean; that is, falling below the interval that encompasses 99.7% of the data in a normal distribution in all replicates. Genes with reduced insertion frequency (IF; growth defect) were those that fell between 2 and 3 SD below the mean (95–99.7% of data). Genes with log_2_[normalized RpK] within 2 SD of the mean were considered non-essential. Those with increased IF (growth advantage) were those genes with log_2_[normalized RpK] greater than 2 SD above the mean. As transposon insertion in essential genes may be lethal, genes that lacked transposon insertions were considered essential. Genes with different essentiality calls between replicates were designated “Uncertain.”

### Data Availability

The raw genome sequencing reads (Illumina and NanoPore) have been deposited in the NCBI Sequence Read Archive (SRR18508951 and SRR18508952). The accession number for the genome assembly is GCA_002085925.2. Tn-Seq raw reads for each library are available from BioProject PRJNA821218.

## Results and Discussion

### Genomics

#### General Features of the Genome

ElOx9^T^ was isolated from Catalina Harbor (California, United States) sediments via enrichments on artificial saltwater agar plates supplemented with elemental sulfur/thiosulfate and nitrate as electron donor and acceptor, respectively, and was established as the type strain of *T. electrotropha* sp. nov. (Chang et al., 2018). ElOx9^T^’s genome encodes a 4.27 Mb chromosome and 128 kb plasmid (summary statistics are shown in Table 1). The chromosome has a GC content of 63.87% and encodes 4,014 genes, 52 tRNAs comprising all 20 amino acids, and three rRNA operons. The plasmid has a GC content of 59.98% and encodes 125 genes, including 30 hypothetical proteins and 26 predicted transposases.

**TABLE 1:** Genome assembly and quality statistics for *Thioclava electrotropha* ElOx9^T^.

	Chromosome	Plasmid
Genome completeness (%)^a^	99.39	N/A
Est. contamination (%)^a^	0.15	N/A
Quality^b^		
# contigs	1	1
Largest contig (bps)	4,267,812	127,662
Total length (bps)	4,267,812	127,662
GC (%)	63.87	59.98
# Mismatches (N characters)	0	0
Features^c^		
CDS	4,014	125
With assigned function	3,470	95
Hypothetical	544	30
tRNAs	52^d^	0
rRNA operons	3	0
ncRNA	3	0
CRISPR arrays	0	0
Pseudogenes	72	18
Signal Peptides^e^		
Genes with Sec/SPI signal peptides^f^	383	7
Genes with Sec/SPII signal peptides^g^	89	2
Genes with Tat/SPI signal peptides^h^	72	1

*^a^Determined with CheckM v1.0.18 (Parks et al., 2015).*

*^b^Determined with QUAST v4.4 (Gurevich et al., 2013).*

*^c^Determined with the NCBI Prokaryotic Genome Annotation Pipeline (Tatusova et al., 2016; Haft et al., 2018).*

*^d^Comprises all 20 amino acids.*

*^e^Determined with SignalP 5.0 (Almagro Armenteros et al., 2019).*

*^f^Sec/SPI denotes signal peptides transported by the Sec translocon and cleaved by Signal Peptidase I (Lep).*

*^g^Sec/SPII denotes lipoprotein signal peptides transported by the Sec translocon and cleaved by Signal Peptidase II (Lsp).*

*^h^Tat/SPI denotes Tat signal peptides transported by the Tat translocon and cleaved by Signal Peptidase I (Lep).*

Taxonomically, the 16S rRNA gene of ElOx9^T^ is most similar to that of *T. nitratireducens* strain 25B10_4 (100% query coverage, > 99% identity, accession no. CP019437.1). Comparisons at the genome level demonstrate greater genetic diversity across the sequenced representatives than is seen at the 16S rRNA level. Whole genome ANI comparisons were made between ElOx9^T^ and closely related draft and complete genomes as identified by GTDB (Supplementary Figure 1). ElOx9^T^ shares 76.2–95.6% ANI with neighboring organisms’ genomes and was most similar to *T. sediminum* TAW_CT_134_^T^ (accession no. GCF_002020355.1), a nitrate-reducing marine bacterium isolated from coastal sediments around Xiamen Island, Fujian province, China (Liu et al., 2017).

#### Metabolic Potential of ElOx9*^T^*

**FIGURE 1:**
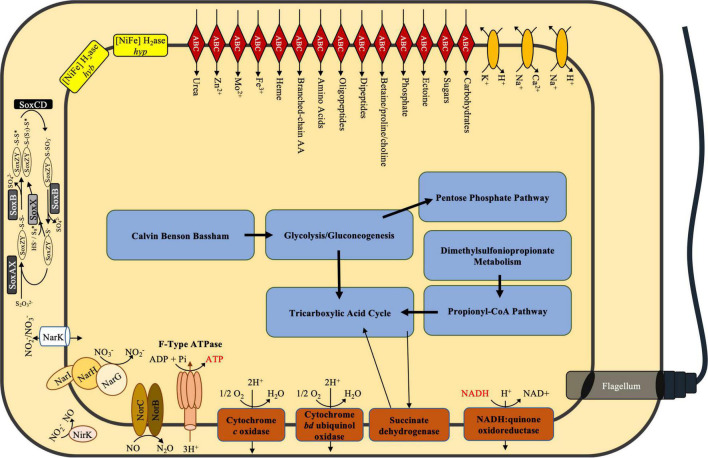
Simplified genome-resolved central metabolic model for *Thioclava electrotropha* ElOx9^T^. See Supplementary Table 2 for a list of genes comprising central metabolic pathways.

ElOx9^T^ is capable of heterotrophic growth on acetate and glucose with either oxygen or nitrate as the terminal electron acceptor, and autotrophic growth with the oxidation of elemental sulfur, thiosulfate, or hydrogen (Chang et al., 2018). Metabolic pathways reconstructed from the genome with BLASTKoala (Kanehisa et al., 2016) corroborate these observations. The genome encodes complete pathways for the Embden–Meyerhof–Parnas (glycolysis), gluconeogenesis, the Entner–Doudoroff pathway, the pentose phosphate pathway, the citric acid cycle, and the reductive pentose phosphate pathway (Calvin Benson Bassham cycle). ElOx9^T^’s genome lacks isocitrate lyase but encodes malate synthase. Thus, this organism may be incapable of bypassing the decarboxylation steps of the TCA cycle via the glyoxylate shunt and may form malate from glyoxylate. ElOx9^T^ has demonstrated growth with acetate as the sole carbon source, likely via conversion to acetyl-CoA by acetyl-CoA synthase, rather than via the glyoxylate cycle. A complete pathway for oxidative phosphorylation and an incomplete denitrification pathway – lacking nitrous oxide reductase *nosZ* – were annotated. Genomic evidence for lithotrophic metabolisms includes the complete sulfur oxidation pathway (*soxABCDXYZ*) and [NiFe] hydrogenase gene clusters (metabolic potential overview in Figure 1 and Supplementary Table 2).

Complete amino acid biosynthesis pathways for all 20 amino acids were identified, thus auxotrophies are not predicted and have not been observed in culture (Chang et al., 2018). The genome encodes 76 putative carbohydrate-active enzymes (Supplementary Figure 2), primarily glycosyl transferases and glycoside hydrolases. Some of these proteins include mannitol 2-dehydrogenase, phosphomannomutase, α- and β-glucosidases, α-galactosidase, in addition to several proteins putatively involved in the degradation of cell wall components. The genome encoded 231 peptidases and 86 non-peptidase homologs, and 12 peptidase inhibitors (Supplementary Table 3). The most numerous peptidase families were: family S33 prolyl aminopeptidases, which preferably cleave N-terminal proline residues, thus providing the organism with proline-rich substrates; family U69 self-processing peptidases, such as some adhesins, that may autocatalytically cleave the extracellular portion of the protein upon translocation to the outer membrane, which aids in adhesion to surfaces; family M23B [8] beta-lytic metallopeptidases, such as lysostaphin, which act on cell wall peptidoglycan and are often used in defense or feeding; family M20 and subfamily M20D exopeptidases, including carboxypeptidases and dipeptidases that primarily function in facilitating intracellular protein degradation and recycling; and family C26 gamma-glutamyl hydrolases that act on gamma-linked glutamate bonds in peptides. These predictions suggest that ElOx9^T^ is metabolically versatile with respect to autotrophic and heterotrophic growth, but also may be capable of metabolizing a variety of organic substrates.

ABC transporters for the following compounds were predicted from the genome sequence: rhamnose, D-ribose, c4-dicarboxylate, and other sugars and carbohydrates, ectoin/hydroxyectoin, glycine betaine/L-proline, choline, taurine, thiamine, lipoprotein, oligopeptides, phosphate, phosphonate, urea, heme, molybdenum, zinc, and other metals. The several glycine-betaine/L-proline ABC transporters present in the genome are of particular interest as they have been known to transport dimethylsulfoniopropionate (DMSP; Cosquer et al., 1999), a major organic sulfur compound produced by marine phytoplankton (Yoch, 2002). A metagenomic study of seawater and sediment samples collected along a 10.9 km depth profile in the Mariana Trench identified high abundances of cells containing DMSP catabolic genes belonging primarily to several genera of Actinobacteria, Gammaproteobacteria, and Alphaproteobacteria, including *Thioclava* (Zheng et al., 2020). ElOx9^T^ has the genomic potential to utilize DMSP as a carbon and energy source, in a process that would release the greenhouse gas dimethylsulfide (Lovelock et al., 1972).

#### Identification of Putative Redox-Active Proteins in the Cell Envelope

Though ElOx9^T^ has demonstrated capacity for oxidative EET, the genes responsible for conferring this capacity have yet to be identified. The primary characterized mechanism of direct extracellular electron transfer in model organisms such as *S. oneidensis* MR-1 and *Geobacter* species – both primarily known for their mineral reduction metabolisms – entail the use of outer membrane multiheme *c*-type cytochromes (Paquete et al., 2022). ElOx9^T^ lacks genes with homology to either the Mtr pathway in MR-1 or the outer membrane cytochromes in *G. sulfurreducens.* As ElOx9^T^ lacks genes canonically involved in anode respiration in model organisms, we sought to identify putative redox active proteins that contain heme-binding motifs (CX_2–4_CH). Of the 54 heme-binding-motif-containing proteins identified, 33 were predicted to localize to the cell envelope, including 7 to the cytoplasmic membrane, 10 to the periplasm, and 16 with unknown localizations (Supplementary Table 4). The majority of these proteins contain a single heme-binding motif and the maximum number of motifs identified was 8 in a tetrathionate reductase family octaheme *c*-type cytochrome (AKL02_08055). Complicating the analysis of putative heme-binding-motif-containing proteins is the absence of distinct functional annotations. Therefore, to determine if any of these proteins are involved in oxidative EET, we employed Tn-seq methods to identify essential genes under electrotrophic conditions.

### Essential Gene Analysis via Transposon Insertion Mutagenesis Screens

#### Construction and Selection of Complex Transposon Mutant Library in ElOx9^T^

To create a transposon mutant library, the mini*Himar1* transposon from an *E. coli* WM3064 strain harboring the pMini*Himar* RB1 transposon delivery vector (Bouhenni et al., 2005) was conjugated into ElOx9^T^ (Chang et al., 2018) as outlined in Methods. This parent library was outgrown in LBS + Ions semi-rich media under aerobic conditions, which generated in excess of 91,000 transposon insertion mutants covering > 69,000 individual TA dinucleotide sites throughout the genome. This parent library was then subjected to differential growth conditions, including aerobic heterotrophic growth in minimal media (SWB) with acetate, and autotrophic oxidative EET conditions (SWB), with electrochemistry pre-growth (Pre-Electrochemistry) and open circuit conditions serving as controls for growth on minimal media and attachment phenotypes, respectively. All conditions were tested in at least triplicate (quadruplicate for oxidative EET). Heterotrophic conditions were monitored by measurement of optical density, ensuring a minimum outgrowth of six generations in each replicate. Cells from overnight growth in SWB + acetate, monitored by taking OD_600_ measurements, were used to inoculate bioelectrochemical reactors and aliquots were taken for the Pre-Electrochemistry Tn-seq control condition. Electrochemistry replicates were monitored for maximal current production during chronoamperometry at –278 mV vs. SHE (approximately 5 days, Supplementary Figure 4). Cyclic voltammetry was performed on all replicates to verify the current generation was consistent with previously characterized ElOx9^T^ electrochemistry, as kill controls were not feasible with this experimental design (Supplementary Figure 4).

#### Transposon Insertion Mutagenesis Screens Sequencing Library Statistics

In all libraries, most quality-controlled sequencing reads aligned to the genome a single time (73.9–90.4% mapping frequency, Supplementary Table 5). Greater than 90% of the unique insertion sites identified in each technical replicate (LBS + Ions parent library) were identified in all replicates of the same growth condition, suggesting that insertion sites identified by our approach are primarily authentic. In fact, we observed high concordance among all replicates within each growth condition supporting the reproducibility of our approach (Supplementary Table 5).

The mini*Himar1* transposon preferentially inserts at TA dinucleotide sequences (Lampe et al., 1998; Yang et al., 2014). *in silico* analysis identified 129,162 potential mini*HimarI* transposon insertion sites within ElOx9^T^’s genome. Individual library coverage percentages ranged from 45.7 to 56.7% of total TA sites (Supplementary Table 5). While insertion densities below 100% are not unexpected, other investigations employing *mariner* transposon-based Tn-seq methods calculated an insertion density of 83% (Perry and Yost, 2014). It is conceivable that our lower transposon insertion frequency may be due to sequencing reads mapping to non-unique TA sites throughout the genome. In each library, approximately 5–7% of quality-controlled reads are aligned to multiple TA dinucleotide sites. Considering the expected genomic DNA insert length was 14 base pairs given our experimental design and the average genomic DNA insert length was ∼14.5 bp for all libraries (Supplementary Table 5), we calculated the number of duplicated TA sites (14bp), which equated to 11,864 sites throughout the genome. Despite this caveat, sequencing reads mapped to 4,024 genes in ElOx9^T^’s genome. After exclusion of 33 genes lacking TA sites entirely (Supplementary Table 6), 10 genes lacking TA sites when ignoring insertion sites in the last 5% of the gene sequence (Supplementary Table 7), and 35 genes lacking unique TA sites in the first 95% of the gene (Supplementary Table 8), gene coverage is estimated at 98.8%.

#### Identification of Essential Genes

Pairwise comparisons of log_2_[normalized RpK] values for all replicates within a growth condition (Supplementary Table 9) revealed strong correlations between replicates (*R^2^* > 0.97 for most comparisons). For each replicate, log_2_[normalized RpK] values were analyzed via histogram. Outliers were removed and the mean and standard deviation of the distribution was calculated. A normal distribution curve was fitted to the data using the mean and SD (Supplementary Figure 3). Essential genes were those with log_2_[normalized RpK] values less than 3 SD below the mean in all replicates; genes with a growth defect had reduced insertion frequency that fell 2–3 SD below the mean; genes considered non-essential fell within 2 SD of the mean, and genes with a growth advantage had increased insertion frequency that was greater than 2 SD above the mean (Supplementary Tables 10–15). Insertions in rRNAs and tRNAs, genes lacking insertions in unique TA sites, and genes for which essentiality calls differed among all replicates in a growth condition were excluded from our analysis. It is important to note that this stringent statistical method for defining essentiality may result in the misidentification of a small proportion of genes as non-essential. Nonetheless, we believe our approach, rigorous analysis, and demonstrated replicability provide an accurate and conservative prediction of gene essentiality.

#### Core Essential Genome

**FIGURE 2:**
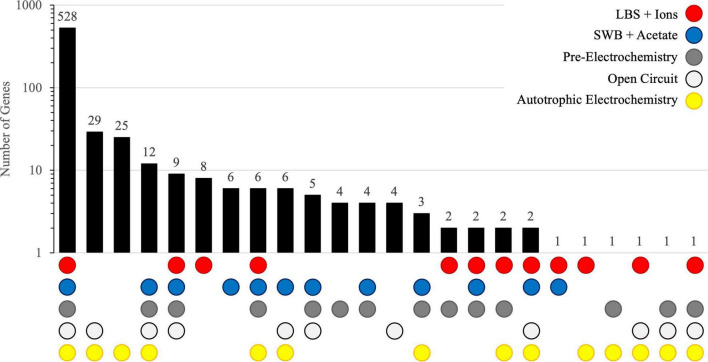
UpSet plot showing the distribution of essential genes identified by Tn-seq under all five growth conditions (LBS + Ions – red circle, SWB + Acetate – blue circle, Pre-Electrochemistry (pre-growth in SWB + Acetate) – gray circle, Autotrophic Open Circuit condition – white circle, and Autotrophic Electrotrophy – yellow circle). Note: y-axis is log scale.

The distribution of gene essentiality calls for all libraries is shown in Table 2. Most of the essential genes identified in each library were classified as essential in all libraries (Figure 2 and Supplementary Table 16). These 528 essential genes comprise the *core essential genome* of ElOx9^T^ and represent 12.7% of the genome. Previous Tn-Seq studies in Bacteria show the percentage of open reading frames deemed essential ranges from just 5% in *Burkholderia cenocepacia* (Wong et al., 2016; 8.06 Mb genome) to 80% in *M. genitalium* (Glass et al., 2006; 0.58 Mb genome). The majority of the essential genes (82%) have KEGG ontology (KO) annotations and are predicted to be involved in many functional categories, including carbohydrate metabolism (primarily glycolysis, TCA cycle, and PPP); oxidative phosphorylation; biosynthesis of lipids, nucleotides, amino acids, aminoacyl-tRNAs, peptidoglycan, cofactors, vitamins, and porphyrin; genetic information processing (DNA replication and repair, transcription, translation, and protein folding and sorting), environmental sensing (transport and secretion[*sec* and *tat*]), and cellular processes (cell cycle and division). The remaining essential genes without KO annotations were comprised primarily of hypothetical proteins (40 in total), proteins containing domains of unknown function, other proteins with poorly characterized function, transcriptional regulators, and stress response. Unsurprisingly, a similar set of essential genes was identified via Tn-seq in the metabolically versatile Alphaproteobacterium *Rhodopseudomonas palustris* (Pechter et al., 2016).

**TABLE 2:** Distribution of gene essentiality calls for all libraries.

	Essential	Growth defect	Non-essential	Growth advantage	Uncertain
LBS + Ions	563	105	3,270	15	108
SWB + Acetate	584	105	3,249	10	113
Pre-Electrochemistry	580	99	3,250	11	121
Open Circuit	598	73	3,188	6	196
Electrochemistry	618	37	3,059	1	346

*The “Uncertain” category represents the number of genes that had discrepancies in essentiality calls between all replicates.*

Ten of the 528 essential genes were located on ElOx9T’s 128kb plasmid and encoded three transposases, two proteins in the type II toxin/antitoxin system, a transcriptional regulator, *repABC* operon involved in plasmid replication and segregation, and several hypothetical proteins. *repABC* plasmids are common among the Alphaproteobacteria (Cevallos et al., 2008). The presence of these features in the essential genome suggests that the plasmid is an indispensable genetic element, however, the role of plasmid-encoded genes in the growth and survival of ElOx9^T^ is not immediately clear and warrants further study.

#### Essential Genes Under Heterotrophic Conditions

The essential gene profiles for growth under heterotrophic conditions (LBS + Ions, SWB + acetate, and Pre-Electrochemistry) were remarkably similar. Eight genes were identified as essential in LBS + Ions only, eight in all conditions except LBS + Ions, 6 solely in SWB + acetate, and 4 in Pre-Electrochemistry (Figure 2 and Supplementary Table 16). Most of these essential genes, regardless of growth condition, are predicted to be involved in major carbon metabolism pathways, such as the TCA cycle, glycolysis, and biosynthetic pathways. Despite seemingly incongruent essentiality calls for these genes in libraries grown under aerobic heterotrophic conditions, all but one of the genes identified as essential in one condition were designated essential, growth defect, or uncertain in all other conditions. That is, for all the genes deemed essential in at least one heterotrophic growth condition, only one of these genes was classified as non-essential in another growth condition. This gene, a DUF1643 domain-containing hypothetical protein (AKL02_18975), was essential or uncertain in all conditions except in LBS + Ions, where it was designated non-essential. BLASTP searches show that this protein is found in a variety of *Thioclava* species and organisms in closely related genera, although the role of DUF1643 remains unknown (Mir-Sanchis et al., 2016).

#### Oxidative Extracellular Electron Transfer in ElOx9^T^ Does Not Use the Sox System or Hydrogenases

Chemolithoautotrophic growth with thiosulfate, S^0^, and H_2_ serving as electron donors has been demonstrated in ElOx9^T^ (Rowe et al., 2015; Chang et al., 2018). In bioelectrochemical investigations, electrodes are often poised at environmentally relevant redox potentials to mimic those of solid-phase minerals, effectively serving as non-corrodible proxies for minerals. In the present experiment, the working electrode was poised at –278 mV vs. SHE, just below the calculated redox potential for S^0^ oxidation in artificial SWB at pH 8 (Rowe et al., 2015), which should provide the organisms with even more reducing power compared to S^0^. To our surprise, Tn-seq revealed that *soxA*, *soxB*, *soxC*, *soxD*, *soxX*, *soxY*, and *soxZ* were non-essential for growth under any of the conditions tested. Similarly, with a single exception – a HypC/HybG/HupF family hydrogenase formation chaperone AKL02_15805 that was essential under all conditions tested – genes involved in hydrogenase biosynthesis were non-essential. This may seem unexpected, as the Mtr pathway in *S. oneidensis* MR-1 is required for both Fe^3+^ and Mn^4+^ reduction and anode reduction (Beliaev and Saffarini, 1998; Beliaev et al., 2001; Bretschger et al., 2007). This indicates that neither the *sox* system nor hydrogenases are essential for oxidative EET in ElOx9^T^ under the conditions tested. Interestingly, another *Thioclava* species, *T. dalianensis* DLFJ1-1^T^ (Zhang et al., 2013) has the capacity for electrode oxidation (Chang et al., 2018) but lacks the ability to oxidize reduced sulfur compounds (Liu et al., 2015). If electrode oxidation is a common feature among this genus and is evolutionarily conserved, these observations suggest that it is not unexpected that the *sox* system is not essential for oxidative EET and indicates some other mechanism is responsible for this phenotype.

#### Essential Genes for Extracellular Electron Transfer

Initially, open circuit control conditions were included to differentiate genes required for biofilm formation and attachment from those directly involved in EET. However, recent evidence has shown that microbial colonization on non-corrodible conductive materials (electrodes used in bioelectrochemical investigations) by organisms with EET capacity was responsible for a shift in open circuit potential by up to 150 mV, suggesting that conductive materials may be serving as conduits for EET even without an applied potential (Bird et al., 2021). Although open circuit potentials were not measured for our open circuit conditions, given the observation that some electroautotrophs are capable of catalyzing a shift in open-circuit potential, we chose to consider the 29 essential genes found in both open circuit and electrochemical conditions as putatively essential for EET in ElOx9^T^ alongside the 25 genes deemed essential under oxidative EET conditions alone. Nearly all genes deemed essential for oxidative EET were non-essential in all other conditions (excluding open circuit) and only five genes essential for both oxidative EET and open circuit were non-essential in all other conditions (Supplementary Table 16).

Unexpectedly, just one of these genes contains a putative heme-binding motif: AKL02_00085, a NAD(P)-dependent oxidoreductase predicted to localize in the cytoplasm. Known direct electron transfer mechanisms involve multiheme cytochromes localized to the outer membrane, in pili or nanowires, or contained in outer membrane vesicles [reviewed in (Paquete et al., 2022)]. To that end, just fourteen of the essential genes were predicted to localize to the cell envelope (Table 3). As we are most interested in genes involved in extracellular electron transfer, we focus our discussion on a few of these proteins predicted to facilitate potentially relevant redox reactions. Rubrerythrin family proteins (AKL_00835) are non-heme diiron proteins commonly found in anaerobic bacteria, some of which use electrons from NAD(P)H to reduce H_2_O_2_ to H_2_O and alleviate oxidative stress (Caldas Nogueira et al., 2021). AKL02_16390 encodes a quinone-dependent dihydroorotate dehydrogenase. Dihydroorotate dehydrogenases are involved in pyrimidine biosynthesis but are also involved in energy metabolism. These flavin mononucleotide-containing enzymes facilitate the oxidation of dihydroorotate to orotate and transfer those electrons to quinones (Sousa et al., 2021). AKL02_07710 is annotated as a DUF59 domain-containing protein and BLASTP indicated this protein in ElOx9^T^ has nearly 100% homology to SUF system Fe-S cluster assembly proteins found in a variety of *Thioclava* species. Although the function of many DUF59 domain-containing proteins has yet to be determined, DUF59-containing SufT in *S. aureus* and *Sinorhizobium meliloti* have roles in iron-sulfur cluster assembly [reviewed in (Mashruwala and Boyd, 2018)]. Hypothetical protein AKL02_14895 lacks any biochemically characterized homologs, but pSORTb predicted a cytoplasmic membrane localization and InterProScan predicted a non-cytoplasmic region flanked by an N-terminal transmembrane helix and a C-terminal transmembrane region. Finally, AKL02_06520 is annotated as NADH:ubiquinone oxidoreductase subunit NDUFA12, a component of the mitochondrial respiratory chain complex I. In ElOx9^T^, this gene is located in an operon with outer membrane lipid asymmetry maintenance protein mlaD (AKL02_06525, non-essential) and a DUF2155 domain-containing protein (AKL02_06530, non-essential). NDUFA12 is commonly found either in a domain fusion or a gene neighborhood with mlaD in the Alphaproteobacteria (Isom et al., 2017) and is theorized to form a supramolecular complex spanning both inner and outer membranes (Ekiert et al., 2017). The role of these proteins in EET cannot be directly discerned from Tn-seq data. In this instance, the utility of the Tn-seq approach was to identify genes which, when disrupted by transposon insertion, caused a significant deleterious effect on growth. Tn-seq successfully identified a short list of genes for investigating the mechanism of oxidative extracellular electron transfer in ElOx9^T^.

**TABLE 3:** Essential genes in electrochemistry or electrochemistry/open circuit conditions predicted to localize to the cell membrane or of unknown localization.

Locus tag	Product	KEGG Orthology	Localization (Score)	Homologs identified in other electrotrophic *Thioclava* species^1^
AKL02_09955	HlyC/CorC family transporter	–	Cytoplasmic Membrane (10)	A, B, C
AKL02_00835	rubrerythrin family protein	K22737	Cytoplasmic Membrane (10)	A, B, C
AKL02_02090	Na^+^/H^+^ antiporter subunit G	K05564	Cytoplasmic Membrane (10)	A, B, C
AKL02_12420	CDP-diacylglycerol–serine O-phosphatidyltransferase	K17103	Cytoplasmic Membrane (10)	A, B, C
AKL02_06280	ribosome biogenesis GTPase Der	K03977	Cytoplasmic Membrane (7.88)	A, B, C
AKL02_20245	ParA family protein	K03496	Cytoplasmic Membrane (7.88)	A, B, C
AKL02_00700	flagellar basal body-associated FliL family protein	K02415	Cytoplasmic Membrane (9.82)	A*, C*
AKL02_16390	quinone-dependent dihydroorotate dehydrogenase	K00254	Cytoplasmic Membrane (9.82)	A, B*, C
AKL02_11895	ABC transporter ATP-binding protein	K02013	Cytoplasmic Membrane (9.82)	A, B, C
AKL02_14895	hypothetical protein	–	Cytoplasmic Membrane (9.86)	A^, B^, C^
AKL02_10975	PAS domain-containing sensor histidine kinase	K13598	Cytoplasmic Membrane (9.99)	A, B, C
AKL02_06725	biopolymer transporter ExbD	K03559	Unknown (2.5)	A*, C*
AKL02_06520	NADH:ubiquinone oxidoreductase subunit NDUFA12	–	Unknown (2)	A, B, C
AKL02_07710	DUF59 domain-containing protein	–	Unknown (2)	A, B, C

*^1^Electrotrophic Thioclava species: A, T. atlantica 13D2W-2^T^; B, T. dalianensis DLFJ1-1^T^; C, T. indica DT-234^T^. Proteins are considered conserved if BLASTP results indicate > 80% AAI and > 80% query coverage.*

*60–80% AAI with > 80% query coverage.

*^60–80% query coverage with > 80% AAI.*

#### Putative Genes Essential for Extracellular Electron Transfer in ElOx9^T^ Are Found in Other Electroactive *Thioclava* Species

Chang et al. (2018) demonstrated that three previously characterized species of *Thioclava* are also capable of electrode oxidation: *T. atlantica* 13D2W-2^T^ (Lai et al., 2014), *T. dalianensis* DLFJ1-1^T^ (Zhang et al., 2013), and *T. indica* DT-234^T^ (Liu et al., 2015). To evaluate the phylogenetic breadth of putative extracellular electron uptake pathways in *Thioclava* species, we used BLASTP to search the genomes of these three organisms for homologs to the essential EET genes identified in our Tn-Seq screen predicted to localize to the cellular envelope (Table 3). Homologs for all genes were found in 13D2W-2^T^ and DT-234^T^, and homologs for all but two genes were found in DLFJ1-1^T^. The presence of nearly all the essential EET genes in ElOx9^T^ that localize to the cellular envelope in other electroactive *Thioclava* species (Table 3) may hint at a conserved mechanism of oxidative EET in this clade of organisms. Further investigation into the involvement of these genes in oxidative EET and the phylogeny of these proteins will help shed light on this outstanding question.

#### Genes Implicated in Oxidative Extracellular Electron Transfer in *S. oneidensis* MR-1 Are Found in ElOx9^T^, but Are Non-essential

A recent study into the genetic basis of oxidative EET in MR-1 has shed light on the genetic basis of oxidative EET in this organism. Rowe et al. (2021) identified five proteins that, while not essential for oxidative EET, their deletion results in a statistically significant reduction in electron uptake. Given the lack of knowledge on oxidative EET mechanisms, we searched for homologs of the oxidative EET proteins identified in MR-1 in the genome of ElOx9^T^. Four of the five proteins newly identified in the electron uptake pathway in MR-1 have putative homologs in ElOx9^T^ (Supplementary Table 17). AKL02_11685, AKL02_17145, and AKL02_17885 were all deemed non-essential under the growth conditions tested. The essentiality of AKL02_15595 was deemed uncertain under electrotrophic conditions and non-essential under all others; however, this gene was non-essential in three of the four electrochemistry replicates and was classified as a growth defect in the fourth. These data suggest that the oxidative EET genes identified in ElOx9^T^ are non-essential under all conditions tested, but this does not indicate that they do not play a role in oxidative EET. In fact, these five genes were identified in MR-1 by specifically targeting non-essential genes in the genome; they are not essential for oxidative EET in MR-1. This same phenomenon may be true in ElOx9^T^ and the identification of these genes in ElOx9^T^ provides a starting place for investigating the role of non-essential genes in oxidative EET and expanding our knowledge of the diversity of EET mechanisms among Bacteria.

## Conclusion

We employed the Tn-seq approach to glean information on the genetic underpinnings of oxidative extracellular electron transfer in the metabolically versatile Alphaproteobacterium *T. electrotropha* ElOx9^T^ and identified 54 proteins not previously implicated in this process. While the majority of EET essential proteins are predicted to localize to the cytoplasm, a subset localizes to the cell envelope or has unknown localizations. To our surprise, none of the genes identified are multiheme cytochromes. The lack of heme-binding-motif-containing proteins essential for EET may be due to redundant or overlapping functions of multiple proteins involved in the EET pathway or could be due to ElOx9^T^ employing multiple EET pathways altogether. For example, *G. sulfurreducens* employs a variety of mechanisms to transfer electrons to/from the extracellular environment, including conductive pili and periplasmic, inner-, and outer-membrane-bound *c*-type cytochromes, all of which have multiple homologs encoded in the genome (Reguera et al., 2005; Shi et al., 2009; Chan et al., 2017; Uekia, 2021). If ElOx9^T^ employs multiple mechanisms for EET, Tn-seq may not aid in identifying EET genes. Another possibility is that disruption of certain genes involved in EET does not result in a loss of function. Rowe et al. (2021) identified five proteins involved in oxidative EET that were deemed non-essential in Tn-seq screens. All these genes, when individually deleted, resulted in reduced capacity for electron uptake but did not knock out EET capacity entirely. Thus, it is conceivable that genes involved in EET may not be identified as essential unless their disruption results in a highly deleterious or lethal effect on growth. These investigations highlight the potential complexities to be aware of while investigating EET mechanisms in non-model organisms.

## Data Availability Statement

The data presented in the study are deposited in the NCBI Sequence Read Archive repository, accession numbers SRR18508951, SRR18508952, GCA_002085925.2, and PRJNA821218.

## Author Contributions

AR conceived and designed the work. AR and NK contributed to methods development. AR, NK, EL, and JS executed the experiments. JS, AR, TS, and EW analyzed the data. JS drafted the manuscript. All authors revised the manuscript and approved the submitted version.

## Conflict of Interest

The authors declare that the research was conducted in the absence of any commercial or financial relationships that could be construed as a potential conflict of interest.

## Publisher’s Note

All claims expressed in this article are solely those of the authors and do not necessarily represent those of their affiliated organizations, or those of the publisher, the editors and the reviewers. Any product that may be evaluated in this article, or claim that may be made by its manufacturer, is not guaranteed or endorsed by the publisher.
