# Supplementary material for: Genome-Scale Mutational Analysis of Cathode-Oxidizing Thioclava electrotropha ElOx9T
Source: Front Microbiol. 2022 Jun 10;13:909824. doi: 10.3389/fmicb.2022.909824 (PMC9226611; doi:10.3389/fmicb.2022.909824)
Supplement: Supplementary file 1 [file Data_Sheet_1.PDF]

Supplementary Material for

Genome-scale Mutational Analysis of Cathode-oxidizing *Thioclava electrotropha* ElOx9^T

Joshua D. Sackett[1], Nitin Kamble[1], Edmund Leach[1], Taruna Schuelke[2], Elizabeth Wilbanks[2], and Annette R. Rowe[1]

[1]Department of Biological Sciences, University of Cincinnati, Cincinnati, Ohio, USA
[2]Department of Ecology, Evolution, and Marine Biology, University of California, Santa Barbara, Santa Barbara, California, USA

This file includes: Figures S1-S4

See Supplementary Excel file for Tables S1-S17

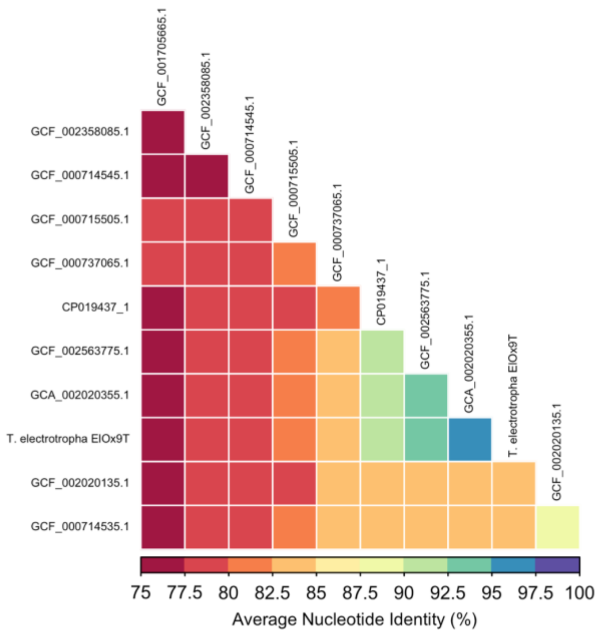

Figure S1. ANI matrix of ElOx9^{T} and closely related genomes identified by GTDB-tk. ANI values ranged from 75.8% to 95.7%. Accession numbers are noted. Note scale bar range of 75-100%.

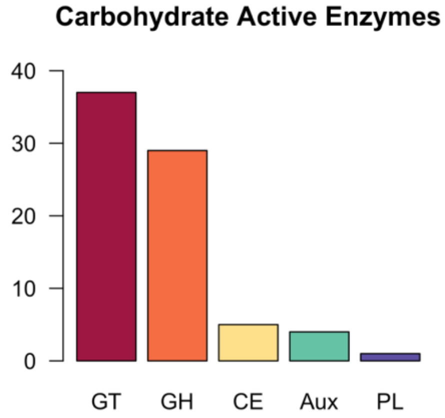

Figure S2. Distribution of carbohydrate active enzymes in the genome of ElOx9^T grouped by enzyme class. GT – Glycosyltransferase, GH – Glycoside hydrolase, CE – Carbohydrate esterase, Aux – Auxiliary activities, PL – Polysaccharide lyase.

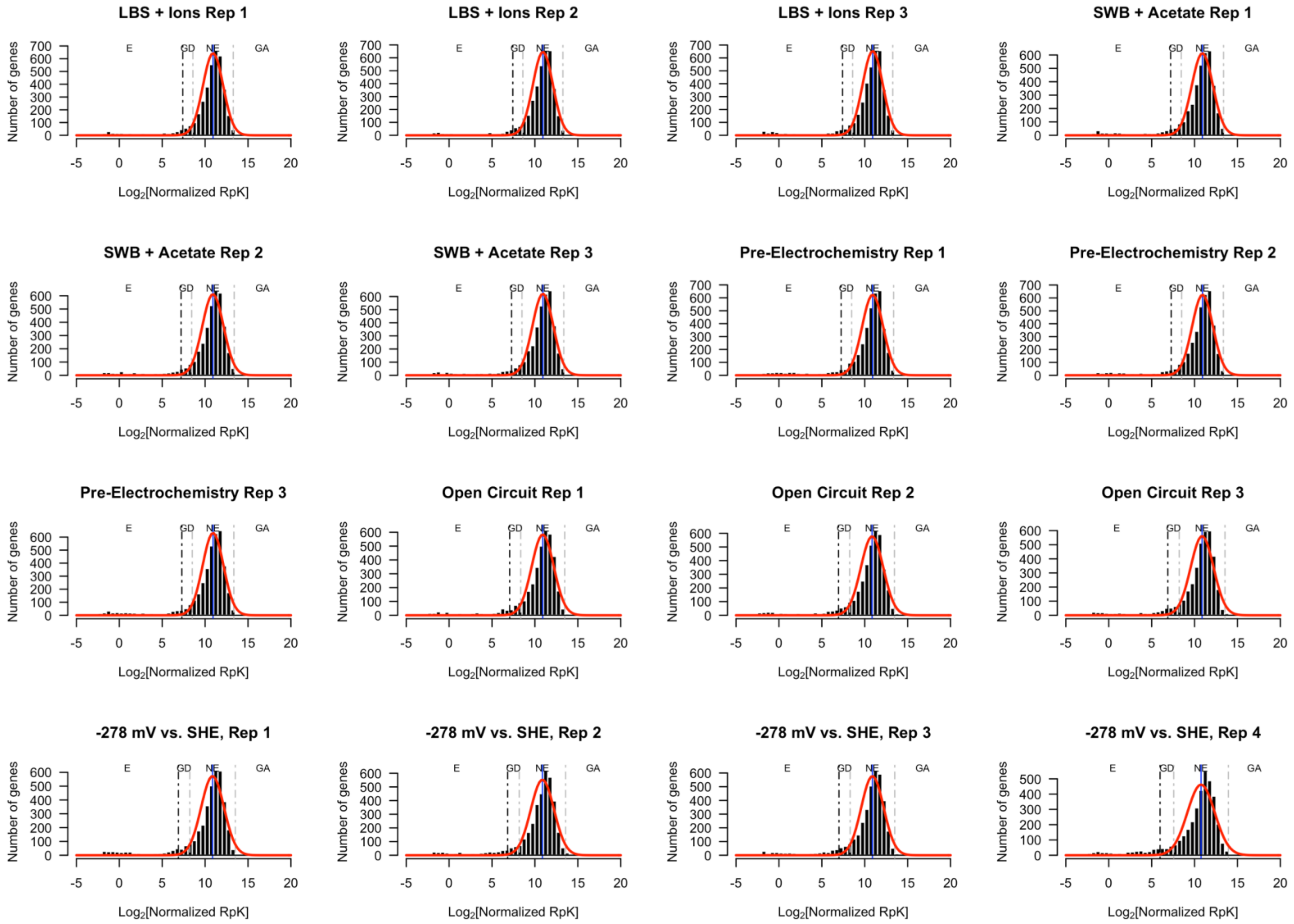

Figure S3. Histograms showing distribution of sequencing depth-normalized RpK values among ElOx9^{T} genes for all growth conditions and all replicates, with the Electrochemistry replicates listed as -278 mV vs. SHE. The means (blue lines) and standard deviations of the distributions were used to generate a normal distribution curve (red line). Essential genes were those with log_2[normalized RpK] values less than 3 SD below the mean (99.7% of the data, 'E', black dot-dash line). Genes with reduced insertion frequency ('GD', growth defect) were those that fell between 2 and 3 SD below the mean (95-99.7% of the data). Genes with log_2[normalized RpK] within 2 SD of the mean were considered nonessential ('NE'). Those with increased insertion frequency ('GA', growth advantage) were those genes with log_2[normalized RpK] greater than 2 SD above the mean. Distances from the mean are indicated by vertical lines on the plot (black dot-dash line, 3SD; grey dot-dash lines, 2SD).

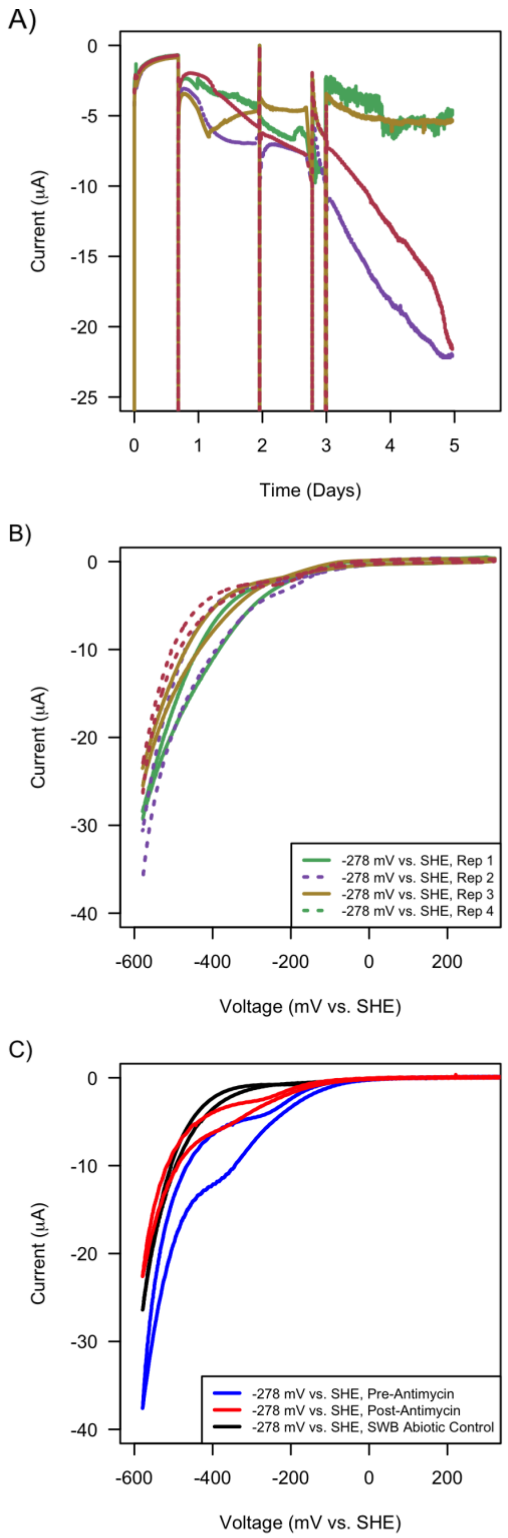

Figure S4. Electrochemical data. (A) Chronoamperometry profiles for all four electrochemistry replicates at -278 mV vs. SHE. The final current achieved in replicates 1-4 was -4.61 µA, -22.1 µA, -5.33 µA, and -21.5 µA, respectively. See panel B for legend. (B) Turnover cyclic voltammetry profiles for all four electrochemistry replicates. (C) Turnover cyclic voltammetry profiles for a separate killed control experiment used to benchmark cyclic voltammograms from Tn-seq experiment. Turnover cyclic voltammetry was performed on cathode-attached *Thioclava electrotropha* ElOx9^T biofilms before (blue line) and after (red line) addition of the ubiquinone mimic Antimycin A (final concentration 50 µM). CV of sterile SWB from the same experiment (black line) is included for comparative purposes.
